# Correlates, Facilitators and Barriers of Healthy Eating Among Primary Care Patients with Prediabetes in Singapore—A Mixed Methods Approach

**DOI:** 10.3390/nu11051014

**Published:** 2019-05-06

**Authors:** Raymond Boon Tar Lim, Wei Keong Wee, Wei Chek For, Jayalakshmy Aarthi Ananthanarayanan, Ying Hua Soh, Lynette Mei Lim Goh, Dede Kam Tyng Tham, Mee Lian Wong

**Affiliations:** 1 Saw Swee Hock School of Public Health, National University of Singapore and National University Health System, Tahir Foundation Building, 12 Science Drive 2, #10-01, Singapore 117549, Singapore; dede146262@yahoo.com (D.K.T.T.); ephwml@nus.edu.sg (M.L.W.); 2 Health Promotion & Preventive Care, National Healthcare Group Polyclinics, 3 Fusionopolis Link, Nexus@one-north, South Tower, #05-10, Singapore 138543, Singapore; wei_keong_wee@nhgp.com.sg (W.K.W.); wei_chek_for@nhgp.com.sg (W.C.F.); jayalakshmy_aarthi@nhgp.com.sg (J.A.A.); ying_hua_soh@nhgp.com.sg (Y.H.S.); 3 Clinical Services, National University Polyclinics and National University Health System, 1 Jurong East Street 21, Singapore 609606, Singapore; lynette_ml_goh@nuhs.edu.sg

**Keywords:** nutrition, public health, prediabetes, primary care, diabetes prevention, mixed methods

## Abstract

Healthy diet remains the primary means to prevent chronic diseases among those with prediabetes. We conducted a mixed methods study, consisting of a cross-sectional survey and in-depth interviews to assess factors associated with fulfilling the healthy plate recommendation, and to explore reasons for the behaviour among primary care patients with prediabetes in Singapore. The prevalence of meeting the recommendation was 57.3%. This was positively associated with being married and negatively associated with being Malay, frequency of eating out weekly and frequency of deep-fried food consumption weekly. The recurrent themes for not meeting the recommendation included family influence, perception of healthy food being not tasty, lack of skills to prepare or choose healthy food, difficulty in finding healthier options when eating out, and healthy food being costly. The recurrent themes for meeting the recommendation included family influence, self-discipline, fear of disease complications, education by healthcare professionals, mass media influence and health promotion campaigns. Much more remains to be done to promote healthy eating among these patients. There were different levels of facilitators and barriers to healthy eating. Apart from the individual and interpersonal levels, practitioners and policy makers need to work together to address the organisational, community and policy barriers to healthy eating.

## 1. Introduction

Prediabetes (intermediate hyperglycaemia) is a high-risk state for diabetes that is defined by glycaemic parameters that are higher than normal, but lower than diabetes thresholds [1]. In individuals aged 45 years, the lifetime risk of progression from prediabetes to diabetes is 74.0% (95% confidence interval, CI 67.6–80.5) [2]. Diabetes is not only associated with all-cause and cardiovascular disease-related mortality [3], its financial burden is also huge [4]. Global spending to treat diabetes and its complications was estimated to be USD 673 billion in 2015 and projected to increase to USD 802 billion by 2040 [4]. The burden of prediabetes and diabetes is disproportionately high in the Asia-Pacific region where more than 60% of the people with diabetes live in Asia [5]. The prevalence of prediabetes is increasing worldwide, and it is projected that more than 470 million people will have prediabetes by 2030 [2]. 

Lifestyle modification remains the primary means to prevent diabetes and cardiovascular diseases among those with prediabetes [1]. Other than regular physical activity, this includes a healthy diet. Among individuals aged 50 to 65 years old, those who eat a healthy diet consisting of whole grains have about a 30% lower risk of developing diabetes compared with those who do not [6]. The 2015–2020 Dietary Guidelines for Americans emphasise the importance of creating a healthy eating pattern to maintain health and reduce the risk of disease such as diabetes, of which MyPlate has been recommended as a simple meal planning tool [7,8]. More details on MyPlate are given in the Section 2.5. Assessment of dependent variable. 

While studies have been conducted to investigate the prevalence and correlates of healthy diet in those with prediabetes, gaps still exist in at least 4 areas [9,10,11,12,13,14]. Firstly, these studies were predominantly from the West rather than in the Asia Pacific region where most people with prediabetes and diabetes reside [9,10,11]. The dietary culture of those in Asia is also different from the West [9,10,11]. For example, bread and potatoes are typical staple food in the West, while this is rice in Asia [15]. Another difference lies in the cooking techniques. Steaming, boiling and stir frying are popular Chinese cooking methods, while pan frying, sautéing and baking are more common in the West [16]. Secondly, these studies focused on different aspects of dietary patterns targeting fruits and vegetables, red meat and carbohydrates consumption rather than MyPlate recommendation. For example, 76% of the participants were not consuming 5 or more servings of fruits and vegetables a day in Canada [9]; while participants consumed red meat with a mean frequency of 3.9 times per week (standard deviation, SD 3.4) in the United States [10]. More information is needed on the prevalence and correlates of individuals with prediabetes who are able to fulfil MyPlate recommendation. Thirdly, most of these studies did not adopt behavioural theories to assess the various influences on dietary behaviour [9,10,11]. Among the few limited studies that had done so, socio-psychological theories at the individual level were employed [12,13,14]. For example, Blue applied the theory of planned behaviour where attitude, subjective norm, and perceived behavioural control were related to the intention of eating a healthy diet [13]. There is a lack of studies which explore the influence of healthy eating at higher levels (e.g., organisational/institutional, community or societal/policy) [12,13,14]. The socio-ecological model (SEM) looks beyond the individual to evaluate multi-level influences, ranging from the surrounding home, work, school, and community environments to social determinants and the influence of public policy on individual behaviour [17]. This has been successfully applied to promote healthy eating among children [18], adolescents [19], elderly [20] and adults living in rural communities [21] but not in individuals with prediabetes, particularly those from the primary care setting. While several trials targeting individuals with prediabetes have successfully demonstrated the impact of healthy eating in preventing progression to diabetes, it is important that such desirable behaviour be sustained beyond the individual level [9,10]. To achieve this, modification at the organisational/institutional, community and policy level is needed other than individual behavioural change. Finally, most of the studies on diet either focused on the facilitators or barriers to healthy eating, but not both [9,10,11]. From the practitioners’ and policymakers’ perspective, it is important to understand both the barriers and facilitators, to better plan programmes for this group. 

The burden of prediabetes and diabetes is high in Singapore with a multi-ethnic distribution [22]. Malays and Indians consistently had higher prevalence of diabetes compared to Chinese from 1998 to 2010 in Singapore [23]. The number of individuals with prediabetes is projected to more than double, from 373,104 in 2010 to 823,802 in 2035 in Singapore [24]. By 2030, 1 in 4 people in Singapore will have prediabetes. Similarly, the prevalence of diabetes, among those aged 20 to 79 years, has been projected to increase from 12.8% in 2014 to 22.7% in 2035 [24]. The total economic cost for working-age patients with diabetes in Singapore was USD 787 million in 2010 and has been projected to rise to USD 1867 million in 2050 [25]. As such, the objective of the study was to assess the correlates of meeting My Healthy Plate (version of MyPlate in Singapore) recommendation [26], and to explore facilitators and barriers relating to the behaviour, among primary care patients with prediabetes in Singapore, using the SEM. More details on My Healthy Plate are given in the Section 2.5. Assessment of dependent variable. The study findings would be useful to inform organisations, communities and policies in planning more tailored dietary interventions for individuals with prediabetes in the Asia Pacific region.

## 2. Materials and Methods 

### 2.1. Study Design

We used the mixed methods approach, comprising a cross-sectional survey (quantitative phase) followed by in-depth interviews (IDIs) (qualitative phase). The explanatory sequential design was used where the first phase involved the collection and analysis of quantitative data, while the subsequent qualitative phase built on these results and explained why they occurred. Based on the quantitative results where participants were categorised according to whether they met My Healthy Plate recommendation, qualitative interviews were conducted sequentially to explore facilitators and barriers for the behaviour in more detail. The purpose of IDIs was to explore the participant’s perspective on the research topic. The participant was considered the expert and the interviewer regarded as the learner. The aim of interview was to learn everything the participant could share about the research topic from his or her world view to gain insights and to better understand the target group [27].

### 2.2. Quantitative Phase

The cross-sectional survey took place in 8 out of 20 polyclinics in Singapore between July 2017 to January 2018. Polyclinics are public healthcare institutions which house primary care doctors and other healthcare professionals such as nurses and dietitians to provide primary care services. They manage about half of the patient population with chronic illness in Singapore [28]. At the start of the study, these polyclinics were managed by 2 public healthcare organisations, of which 1 (National Healthcare Group, NHG) agreed to participate. Out of the 9 NHG Polyclinics, 8 participated while the other one declined due to operational constraints. 

The inclusion criteria for participants of the survey were (i) community-dwelling patients with prediabetes who were Singapore citizens or Singapore Permanent Residents, aged 21 to 79 years, (ii) diagnosis verified by oral glucose tolerance test (OGTT) and diagnosis code, and (iii) currently following up at any one of the 8 polyclinics. At the time of the study, participants who had progressed to diabetes or converted back to normoglycemia based on the last laboratory test and diagnosis code were excluded. The sampling frame came from the polyclinic headquarter database where patients with a diagnosis code of “impaired fasting glycaemia (IFG)” or “impaired glucose tolerance (IGT)” without “diabetes mellitus” were identified. For IFG, the fasting plasma glucose at the point of diagnosis must be 6.1 to 6.9 mmol/L and the 2-hour post OGTT plasma glucose < 7.8 mmol/L. For IGT, the fasting plasma glucose must be 6.1 to 6.9 mmol/L and the 2-hour post OGTT plasma glucose 7.8 to < 11.1 mmol/L. These definitions were consistent with that of the World Health Organisation [29]. Participants were selected through time location sampling at different times of the day at the 8 different polyclinic venues, throughout the operating hours, on weekdays and Saturdays (closed on Sundays). Generally, patients who need to see a healthcare professional or undergo any testing in these polyclinics make prior appointments. Briefly, field recruiters waited at the specific polyclinic according to a pre-determined sampling frame and approached patients who turned up for their appointments. These appointments could be for any reason and need not be prediabetes follow-up. The field recruiter would then invite these patients to participate in the study. If they agreed, the recruiter would obtain informed consent and left them in a quiet corner to complete a survey questionnaire. 

### 2.3. Sample Size Calculation

To detect a prevalence of 60% who reported not meeting the recommendation, with precision of 5% and CI of 95%, would give an estimated minimum sample size of 283. Accounting for a 70% response rate, we aimed to recruit 400 participants for the survey.

### 2.4. Survey Questionnaire

For each participant, the questionnaire was self-administered, with the recruiter being nearby to provide clarification if required. The questionnaire was available in English (Supplementary Materials File S1), Mandarin or Malay depending on the participant’s language preference. The questions were taken from the National Nutrition Survey (NNS) in Singapore [30]. The questionnaire used in the NNS has been administered to the Singapore population since 1993 with several published studies [31,32,33], and hence was familiar to our study population. To minimise social desirability biases, we (i) assured participants that their responses were anonymous, (ii) stressed the importance of responding honestly because their responses would be used for programme improvement, and (iii) ensured that the questionnaire was worded in a non-judgemental manner.

### 2.5. Assessment of Dependent Variable

The variable assessed was whether participants reported meeting My Healthy Plate (version of MyPlate in Singapore) recommendation. According to the 2015–2020 Dietary Guidelines for Americans, MyPlate recommendation consists of 5 food groups [7]. Half of the plate should consist of fruits (whole fruit such as apple, orange and banana) and vegetables (e.g., dark green vegetables such as spinach, broccoli, red or orange vegetables such as tomatoes and carrots, beans and peas), a quarter of the plate should consist of proteins (e.g., seafood, lean meat, poultry and eggs), another quarter grains (preferably whole grains such as wholemeal bread and brown rice) and a smaller circle representing dairy, such as a glass of milk or a yogurt cup [8]. My Healthy Plate is the version of MyPlate adopted for use in Singapore by the Health Promotion Board since 2014 [26]. In contrast to MyPlate, My Healthy Plate recommendation consists of 4 food groups (fruits, vegetables, grains and proteins) without the dairy group [26]. The nutritional recommendation for the 4 food groups are however similar to MyPlate. Fulfilment of My Healthy Plate recommendation was assessed using the question, “Based on your CURRENT diet on a TYPICAL day, are you able to fulfil the following requirements of My Healthy Plate recommended by the Health Promotion Board?” along with an image of the Plate with the relevant description. Participants who reported meeting the recommendation was defined as answering the option of “Yes”, while those who did not was defined as answering the option of “No” or “Not sure” to the question. Participants on special diet (e.g., vegetarian diet) answered the option of “Not applicable”.

### 2.6. Assessment of Independent Variables

Before the study, we reviewed the literature to select the possible factors that could contribute to differences in meeting My Healthy Plate recommendation. These could be broadly categorised into 3 main groups, sociodemographic factors, medical history and dietary habits. Sociodemographic factors included sex, ethnicity, marital status, education level, housing type, current work status and age. Medical history, and dietary habits such as frequency of breakfast, main meals, snacks, eating out, deep-fried food and sweet dessert consumption were also assessed. 

### 2.7. Statistical Analysis 

We obtained the prevalence of participants who reported meeting the recommendation. Those on special diet were excluded from subsequent statistical analysis. Bivariate analysis between meeting the recommendation and each independent variable were carried out. Categorical variables were compared using chi-square test, ordinal variables with the Mann–Whitney U test and continuous variables with the independent-samples t test. We then evaluated the association between meeting the recommendation with each variable using mixed effects Poisson regression model, accounting for clustering by polyclinic venue, to obtain the crude prevalence ratio (PR) and 95% CI. Poisson rather than logistic regression was used as the prevalence of those meeting the recommendation was common (>10% of the study population) [34]. We considered both the statistical and clinical significance when building the multivariable models. To identify the independent factors statistically, those with crude PR of *p* < 0.10 were selected for multivariable analysis. A backward stepwise approach was performed to obtain the adjusted PR (aPR) and 95% CI, where only variables with *p* ≤ 0.05 were included in the final model. For clinical relevance of findings, we reviewed the literature a priori to select the possible sociodemographic, medical and dietary habit factors that could contribute to differences in meeting the recommendation. This also helped to inform the independent variables that were assessed in Section 2.6. In addition, we compared the study sample with the polyclinic headquarter database containing all the NHG Polyclinic patients with prediabetes in terms of sex, marital status, age and ethnicity. All statistical analyses were performed using STATA version 15.0 (Stata Corp, College Station, TX, USA).

### 2.8. Qualitative Phase 

Of the 433 participants who took the survey, 48 underwent IDIs at the National University of Singapore from September 2017 to April 2018. Participants were asked to indicate in the survey questionnaire whether they were keen for the IDIs. Maximum variation sampling strategy was then used to recruit a purposive sample from diverse backgrounds, based on the criteria of sex and whether they reported meeting the recommendation. A priori to recruitment, 4 matrices based on these criteria was created (i. male who reported meeting the recommendation, ii. male who reported not meeting the recommendation, iii. female who reported meeting the recommendation, and iv. female who reported not meeting the recommendation). Participants who had expressed their interest earlier and fulfilled the criteria for each of these matrices were contacted for the IDIs. The interview was conducted using either one of the two topic guides depending on whether they reported meeting the recommendation using the SEM as a framework. This was available in English, Mandarin and Malay, and were pilot-tested before study commencement to ensure coherence and flow of questions. The guide consisted of open-ended questions according to the SEM from individual, interpersonal, organisational, community and policy levels to explore and better understand the participants’ reasons for their behaviour. The interview was conducted in the participant’s preferred language and was audio recorded with consent. All interviews were conducted by the first and seventh authors to ensure consistency. Duration lasted from 30 minutes to an hour. Data saturation was reached.

### 2.9. Qualitative Data Analysis 

The interviews were transcribed verbatim and checked for accuracy against the recordings. Interviews in Mandarin and Malay were translated into English before transcription. These were then imported into NVivo 11.0 and coded line-by-line. The first and seventh authors coded and analysed the data in parallel, independently. We carried out thematic data analysis. This involved reading and re-reading through the transcripts to identify the initial codes. These were then collated into potential subthemes and themes. Several meetings were held to review, define and name the subthemes and themes guided by the SEM. Any discrepancy was resolved by group discussion involving all team members. 

### 2.10. Ethics Approval and Participant Consent

The study was approved by the National Healthcare Group Domain Specific Review Board (approval certificate number 2016/01358). We obtained written consent from all the participants of this study. 

## 3. Results

### 3.1. Quantitative Phase Results 

A total of 433 out of 948 approached were recruited, giving a participation rate of 66.8% for the survey. The main reasons given for non-participation were “busy” and “not interested”. Participants mirrored the total NHG Polyclinic patient pool with prediabetes in sex, marital status and age except for ethnicity. There was a higher proportion of those with other ethnicities in the total NHG Polyclinic patient pool with prediabetes (3.5%) compared to the study sample (1.2%). Out of the 433 participants, 248 (57.3%) reported meeting My Healthy Plate recommendation, 175 (40.4%) did not report meeting the recommendation and 10 (2.3%) were on special diet. Of note, 53.2% of the participants ate out at least 4 times weekly. Table 1 showed the survey participant characteristics (excluding those on special diet). Those who did not report meeting the recommendation were largely similar in sociodemographic characteristics, medical history and dietary habits than those who met except for marital status, age, frequency of eating out and deep-fried food consumption. Those who reported not meeting the recommendation were more likely to be single, younger, ate out more often weekly and consumed deep-fried food consumption more often weekly.

Table 2 showed the crude and adjusted PR of factors associated with meeting the recommendation. Compared with the Chinese, Malays had a lower prevalence of meeting the recommendation (PR 0.81; 95% CI: 0.74–0.89). Married participants had a higher prevalence of meeting the recommendation (PR 1.41; 95% CI: 1.18–1.69) compared to those who were single. Meeting the recommendation was less prevalent among those who ate out more often weekly (PR 0.96; 95% CI: 0.95–0.98), and those with more frequent deep-fried food consumption weekly (PR 0.88; 95% CI: 0.81–0.95). On multivariable analysis, the prevalence of meeting the recommendation was positively associated with being married (aPR 1.38, 95% CI 1.13–1.68) and negatively associated with being Malay (aPR 0.81, 95% CI 0.70–0.95), frequency of eating out weekly (aPR 0.97, 95% CI 0.96–0.98) and frequency of deep-fried food consumption weekly (aPR 0.90, 95% CI 0.82–0.98).

### 3.2. Qualitative Phase Participant Characteristics 

Table 3 showed the IDI participant characteristics. There was no significant difference between those who attended the IDIs than those who did not in terms of sociodemographic characteristics (sex, age, ethnicity, highest education level, housing type and current work status) except for marital status. There was a higher proportion of those who were single among participants who attended the IDIs (25.0%) compared to those who did not (12.5%).

### 3.3. Qualitative Phase Results: Facilitators for Those Meeting the Recommendation 

**Figure 1:**
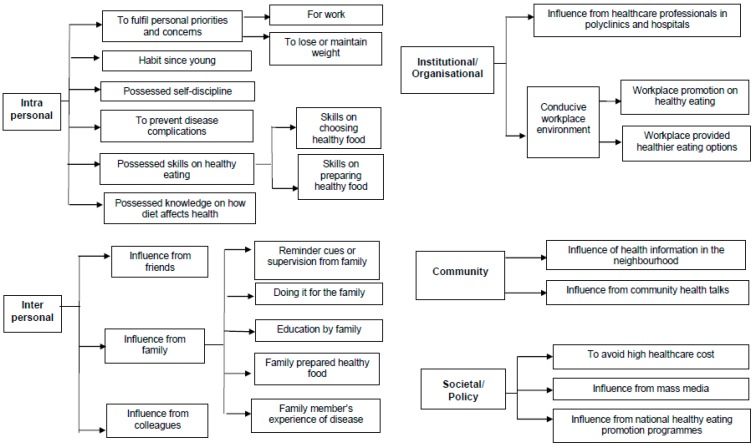
Themes and subthemes pertaining to reasons for meeting My Healthy Plate recommendation.

Figure 1 showed the overview of the themes and subthemes pertaining to facilitators for those who reported meeting the recommendation. 

At the intrapersonal level, participants reported meeting the recommendation as they had knowledge on how diet could affect health, *“I have some knowledge on these diseases. Hypertension, diabetes, kidney problems, generally I know. Unhealthy diet is the main risk factor for these diseases. I know, so I try to avoid and prevent.”* (AMK 054, 67 years old Chinese male). Participants also had the skills on healthy eating such as how to choose healthy food, *“Like coffeeshop, you need to see whether you know how to find healthy food. For example, like the mixed-vegetables stall, try to choose the steamed fish and the vegetables. Don’t eat the vegetables that are stir-fried till oily. Eat the steamed ones, like those cabbage that are cooked with water, that’s healthier compared to the stir-fried ones.”* (TPY 039, 56 years old Chinese male) and how to prepare healthy food, *“Hmm, yes, I can do my salad. I know how to prepare my salad in a healthy way, with different vegetables and fruits, and with minimal seasoning and sauce.”* (AMK 070, 59 years old Chinese female). 

Other participants attributed this to self-discipline, *“I’m a very disciplined person. You can bring me to a buffet lunch or dinner. I don’t eat like everybody else, grab everything you see. I’ll exert self-control and just eat what is healthy, like the vegetables and fruits, rather than the meat.”* (HOU 028, 68 years old Chinese male). For others, it was to fulfil their personal priorities and concerns. Work was one such example, *“One aspect is the need to work. If I am healthy, I can take care of myself and continue to work.”* (AMK 004, 63 years old Chinese female).

The main recurrent themes were observed at the interpersonal level where family influence from spouse and children played an important role in meeting the recommendation. This could be in the form of reminder cues from family, *“...my children will say ‘mom that’s salty, don’t eat’ or you know, they will say ‘this is too fat, don’t eat’, you know what I mean? They will remind me and keep a look-out on my diet.”* (AMK 075, 56 years old Chinese female) or through education by family, *“Like if I buy certain things, my eldest son will teach me to read the food label and tell me, this is not good, artificial, a lot of additives. So, it’s more or less indirectly encouraging me to buy better quality food, more healthy food.”* (AMK 017, 57 years old Chinese female). For some, the family prepared healthy food, particularly the wife or mother in the household. This was highlighted by male participants who were married, *“My wife knows that it is very important for the family to avoid unhealthy food, so I do not need to tell her what to do […] leave it to her. She goes to the wet market or supermarket to buy the more healthy grocery and uses them to prepare our meals.”* (HOU 028, 68 years old Indian male). For others, their family members’ experience of disease motivated them, *“Because I don’t want to end up like my parents, you know, they have high blood, high cholesterol, high sugar. That is why I must control my diet, it is very sad to see how they suffer from these diseases.”* (YIS 005, 57 years old Malay female). 

At the institutional/organisation level, participants reported meeting the recommendation because of influence from healthcare professionals in polyclinics and hospitals, *“The doctor from the polyclinic gives me advice on the percentage of various food that should be on the plate. Like carbohydrate one-quarter, with half a plate of vegetable and fruits and the other quarter is protein. This information is very useful, and I have been doing it since then.”* (CCK 040, 59 years old Chinese male). For others, a conducive work environment facilitated them to meet the recommendation, such as their workplace providing healthier eating options, *“I always eat at my workplace canteen because they serve brown rice. They also have the more healthy options there.”* (WDL 056, 44 years old Indian male). 

At the community level, there was the influence of health information in the neighbourhood, *“The notices in the neighbourhood on living healthy, like those encouraging you to pick up healthy eating are very useful, particularly when you are eating outside. They serve as reminders to me.”* (AMK 075, 56 years old Chinese female). For others, it was the influence from community health talks, *“Because I attend this community health talk, diabetes talk which also teaches you the type of food you are supposed to eat, the healthy plate.”* (AMK 017, 57 years old Chinese female). 

At the societal/policy level, participants reported meeting the recommendation to avoid high healthcare cost, *“I don’t want to fall sick, I don’t want to see doctors so often, so I have to take care of my health. Healthcare cost in Singapore is still too high despite the various subsidies, every time I see a doctor, still have to fork out a certain sum of money, so have to stay healthy by controlling my diet.”* (AMK 004, 63 years old Chinese female). For some, it was due to the influence from mass media, *“Hmm sometimes television programmes. Now they got very interesting programmes, teach you how to cook healthy. Like that Body SOS, Channel 8, that one.”* (AMK 070, 59 years old Chinese female). For others, it was due to influence from the national healthy eating promotion programmes, *“I think the Health Promotion Board has always been promoting healthy living, healthy eating, there was one time when I went for the roadshow and got to be educated on what I am supposed to eat, and what I am not supposed to eat. Then I follow.”* (AMK 005, 63 years old Chinese female).

### 3.4. Qualitative Phase Results: Barriers in Those not Meeting the Recommendation 

**Figure 2:**
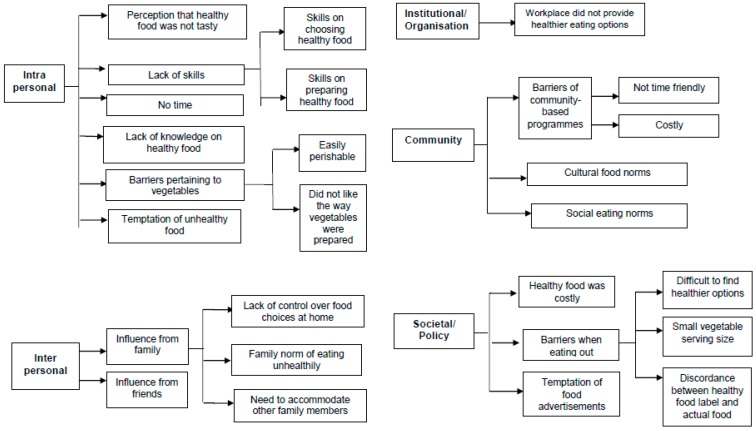
Themes and subthemes pertaining to reasons for not meeting My Healthy Plate recommendation.

Figure 2 showed the overview of the themes and subthemes pertaining to barriers for those who reported not meeting the recommendation. 

At the intrapersonal level, participants did not report meeting the recommendation as they perceived that healthy food was not tasty, *“Because we feel it is really difficult to take the brown rice. Brown rice feels coarser than white rice, it just doesn’t taste good.”* (HOU 041, 46 years old Indian female) or they could not resist the temptation of unhealthy food, *“When we think about the food, we can’t overcome the temptation and so we will give in and eat it. For example, fried food like chicken wings and fast food like burgers, it is just too nice and tempting for me. I just have to take them regularly. If not, I feel something is missing.”* (WDL 052, 62 years old Chinese female). Other participants lacked the knowledge on healthy food, *“No, I don’t know what constitutes a healthy diet, I will just eat whatever I like.”* (YIS 008, 69 years old Malay male). Yet others lacked the skills to choose healthy food, *“I don’t know which one to choose for the vegetables and fruits, which one is healthy, which one not so, which one is suitable for me who have prediabetes.”* (AMK 076, 66 years old Indian male) or to prepare healthy food, *“Because my cooking is all standard, you add the oil, the salt, and the sauce. But if you ask me to cook healthy food, like reduce the oil, reduce the salt, don’t use the sauce, then I don’t know how to cook already. Also, I have been cooking white rice all my life, now you tell me change to brown or red rice, I don’t know how to cook, how to make it tasty like white rice.”* (YIS 002, 57 years old Malay female). There were also barriers pertaining to vegetables such as they were easily perishable, *“So, vegetables cannot put in the fridge you see, very fast will spoil. So, I seldom want to prepare and cook vegetables.”* (AMK 104, 50 years old Chinese female) or they did not like the way vegetable were prepared when eating out, *“It is difficult to get vegetables cooked the way which I like outside. I like my vegetables to be cooked at home.”* (WDL 045, 57 years old Chinese male).

The main recurrent themes were observed at the interpersonal level where family influence played an important role in not meeting the recommendation. Participants expressed that they had no control over food choices at home, *“When I eat at home, I eat whatever my wife cooks, when I come home from work, the food is already cooked so I just eat it. I also cannot control what she cooks, because at home she will decide what to buy and what to cook.”* (WDL 048, 77 years old Chinese male). For some, they need to accommodate other family members, *“For health reason, brown rice is better, but no choice, if you put brown rice then it is only for me to eat. My children all don’t eat, my wife doesn’t eat.”* (AMK 076, 66 years old Indian male). For others, it was the family norm of eating unhealthily, *“My whole family eats white rice since young, it has become a habit, a culture in us. Now say change to brown rice, not easy, it takes time for us to adjust to the new taste of brown rice.”* (HOU 035, 65 years old Chinese female). The influence of friends was important for some, *“…because you have to maintain your social life. You cannot say to your friends, I cannot eat, I only take healthy food nowadays, that’s anti-social and unfriendly.”* (CCK 039, 55 years old Chinese male).

At the institutional/organisation level, participants did not report meeting the recommendation because their workplace did not provide healthier food options, *“There is only white rice available at the workplace canteen, and you don’t find any brown rice or wholegrain. So, I have to eat white rice.”* (TPY 044, 53 years old Chinese female). At the community level, participants experienced barriers pertaining to community-based programmes, including their not being time-friendly, *“Sometimes I want to join some activities at the community centre, like for example there was one on healthy diet, but it was in the morning like 9am and it’s our working hours.”* (HOU 008, 66 years old Chinese female). The cultural food norm was important for some, particularly the Malays, *“Malay food like beef rendang, nasi lemak, biryani, curry chicken, these are usually found in our family gatherings, festival occasion gatherings and wedding banquets. We also eat these at home too. These foods are usually deep-fried and prepared with coconut milk, if not lots of sauces and gravy. I seldom see vegetables or fruits being taken with these foods. Even if there are, it will not fulfil the healthy plate requirement because the rice and meat will take up almost the whole plate.”* (WDL 068, 55 years old Malay female). For others, the social eating norm was highlighted, particularly among the Malays, *“We also have a lot of family and friends gathering, festive gatherings and wedding banquets to attend. In these occasions, it is very difficult to eat healthily because everyone will be taking the white rice, curry, the food cooked with coconut milk. It is standard for us to eat these. This is a part of us since we all like to eat these not only at the gatherings, but also at home. It is very weird for anyone to ask for brown rice or healthier food options in these gatherings, it would also appear that you are not part of the community.”* (YIS 002, 57 years old Malay female). 

At the societal/policy level, participants found healthy food to be costly, *“Yes, the price of healthy choice is a form of deincentivisation to consumers [...], you choose to eat healthily, but you are penalised by the price…”* (AMK 100, 50 years old Chinese male). Others faced barriers, such as difficulty in finding healthier options, *“I mean, most of the hawker stalls, what they offer is minimum in vegetables. You also don’t see stalls that sell fruits or salads. Most of the food they offer is high in carbo and some protein. The fibre in hawker food is usually not enough.”* (TPY 022, 56 years old Chinese male), and small vegetable serving sizes, *“Like I say, you eat outside, you order the vegetables, they give so little. Then most of the food that you order from the hawker centre or coffeeshop also do not come with vegetables.”* (WDL 045, 57 years old Chinese male). The temptation of food advertisements in the mass media was highlighted by some, *“There are a lot of advertisements on food, a lot of promotion especially fast food on TV, then a lot of nice photos […] these are tempting to the public. So being human, once you are hungry, you will be tempted, and you will lose your motivation.”* (CCK 039, 55 years old Chinese male).

## 4. Discussion

Close to 3 in 5 primary care patients with prediabetes reported meeting My Healthy Plate recommendation. This was more prevalent among those who were married, and less prevalent among those who were Malays, those who ate out more often weekly, and those with more frequent deep-fried food consumption weekly. Facilitators and barriers to healthy eating were found at multiple levels, with family influence as a major recurrent theme. 

Our proportion (57.3%) was at the higher end of the range compared to other studies where the prevalence of healthy diet varied from 23.5% to 59.0% [9,10,11,35,36]. Other than differences in the study population, sampling technique, study design, another possible explanation could be differences in the assessment of healthy diet. Some studies used more detailed dietary assessment tools such as the 24-hour dietary recall method [35] or the modified version of the National Cancer Institute diet history questionnaire [10] to assess adherence to a healthy diet. None of the studies used objective assessment such as nutritional biomarkers. Due to the busy nature of the primary care setting, we used a more subjective shorter measurement by showing an image of My Healthy Plate with the relevant description. 

Married individuals were more likely to report meeting the recommendation compared to those who were single. The IDIs suggested that this could be due to family influence, particularly spousal influence; it was usually the wife in the household who decided on the groceries to buy and the food to be served during mealtimes. Previous research in the West also found that spouses played an influential role in their partners’ health behaviours, particularly for dietary behaviour [37]. In the Asian setting, it is common for a married couple to share a meal together [38] and hiding dietary behaviours from a spouse is difficult [39]. Leveraging on the family to promote healthy eating is appropriate in an Asian population, where the family is often regarded as the basic societal unit [40]. From the IDI results, family could exert a positive or negative influence on healthy eating. Therefore, other than focusing on patients with prediabetes, their family members should also be engaged as agents of change. As a start, health promotion messages on healthy eating, targeting those with prediabetes, could incorporate the theme of “eat more healthily with your family”. More family-based healthy eating promotion activities could also be organised in the primary care setting, workplaces and the community.

Compared to the Chinese, Malays reported a lower prevalence of meeting My Healthy Plate recommendation. The IDIs suggested that cultural food norms and social eating norms would impact healthy eating in the Malay community. Abdullah and colleagues reported that Malay adolescents had significantly greater preference for local-based and Western-based food patterns, whereas Chinese adolescents had higher preference for a healthy-based food pattern in Malaysia [41]. Local-based food pattern was defined in the study as a diet consisting of mainly white rice, condensed sweetened milk, tea, seasoning fish sauce “budu”, banana fritters and white bread [41]. Our results suggested that cultural and community norms played an influential role in the dietary behaviour of the Malay community. Further studies are needed to investigate how socio-cultural factors affect food preferences and choices among the Malays to better inform the development of culturally-sensitive healthy eating programmes for this community. 

Meeting the recommendation was negatively associated with those who ate out more often weekly. Consistent with a systematic review, eating outside of the home was associated with a higher total energy intake and energy contribution from fat in the daily diet [42]. Two large studies involving adolescents in United Kingdom and adults in Ireland showed how eating outside of the home was associated with a lower intake of micronutrients, particularly vitamin C, calcium and iron [42]. Both our survey and IDI results revealed that promotion of healthy eating should be extended into the community. Apart from the individual and interpersonal levels, there were various facilitators and barriers to healthy eating at the organisation/institutional, community and societal/policy levels. At the societal/policy level of the SEM, participants expressed several barriers to meeting the recommendation when eating out. Aside from healthy food being costly, these barriers included the difficulty of finding healthier options, the small vegetable serving sizes and the discordance between healthy food labels and the actual food served. Given that 53.2% of the participants ate out at least 4 times weekly (comparable with 60.1% in the general adult population [30]), it is crucial that much more should be done to promote healthy eating outside the home in Singapore. 

Our findings indicated that healthy eating is complex. Future interventions should not only consider intrapersonal and interpersonal influences when aiming to promote healthy eating among this group, but also need to target the organisational, community and policy environments. This means that nutrition promotion strategies for the Asia Pacific region should focus at multiple levels of influence that broadens options for interventions. Policy and environmental changes could potentially benefit a wider population, in contrast to interventions that reach only individuals who choose to participate [43]. In addition, interventions targeting the higher levels of the SEM could potentially foster a more conducive environment to sustain healthy eating, rather than solely relying on intrapersonal factors such as motivation [43]. While education by healthcare professionals in the polyclinics would be useful since they are the first line of contact, our findings have shown that there are various social, cultural and physical barriers to healthy eating that need to be addressed. As such, practitioners and policy makers should consider the SEM when planning nutrition promotion programmes in the Asia Pacific region. This would increase the likelihood of higher-level barriers to healthy eating being addressed. For example, based on our study findings in Singapore, more efforts should be directed towards increasing healthier options, particularly at hawker centres (open-air complexes with many stalls located at the heart of housing estates selling a wide variety of food) and coffeeshops (common eating out venues with a lack of healthier options highlighted by participants) and reducing the cost of healthy food such as whole grains, vegetables and fruits. Collaboration with various food vendors should be stepped up, with a focus on increasing vegetable serving sizes and reducing the discordance between healthy food labels and actual food served. In addition, community organisations and health promotion agencies could collaborate with primary healthcare professionals to change patients’ perceptions of healthy food and also equip patients with healthy food preparation skills. More organisations and settings, such as workplaces, the neighbourhood and the community centres should also be actively engaged to promote healthy eating. 

This study has some limitations. Firstly, during the qualitative phase, we did not show the transcript to the participants to confirm whether their responses had been accurately documented. However, the interviewers mitigated this by regularly paraphrasing and “checking back” with the participants to ascertain the veracity of their responses. Secondly, there was sole reliance on self-reported data of which social desirability bias could not be excluded. To prevent survey fatigue and to ensure reasonable participation, we chose a more subjective shorter measurement rather than the food frequency questionnaire (FFQ) or the Healthy Dietary Index questionnaire. Although steps were taken to reduce the social desirability bias as described in the Section 2.4. Survey questionnaire, further studies are needed to confirm the study findings. Such follow up studies could consider the use of a combination of methods, such as the FFQ with dietary record or the FFQ with biomarker levels to obtain more accurate estimates of dietary intakes rather than relying on an individual method [44]. Thirdly, causal relationships cannot be inferred from the cross-sectional study (quantitative component). Fourthly, although the survey questionnaire (including the assessment of dependent variable) had been pretested, it remained to be validated in Singapore. The questions were however taken from the National Nutrition Survey in Singapore [30], which was familiar to this study population.

Despite the limitations, there were various strengths. This was one of the very few studies to adopt a mixed methods approach in understanding the correlates, facilitators and barriers related to meeting My Healthy Plate recommendation among primary care patients who were at high risk of diabetes. The mixed methods approach enabled triangulation as some of the correlates in the quantitative analysis were also recurrent themes in the qualitative analysis. The IDI findings helped us to better understand the correlates of meeting My Healthy Plate recommendation in the study population. Data saturation was also reached for the qualitative analysis. Although the study sample was not generalisable to all Singapore individuals with prediabetes, it largely mirrored the total NHG Polyclinic patient pool with prediabetes. 

## 5. Conclusions

Much more remains to be done to promote healthy eating among primary care patients with prediabetes seeking care from NHG Polyclinics in Singapore. There were different levels of facilitators and barriers to healthy eating, with family influence as a major recurrent theme. Apart from the individual and interpersonal levels, practitioners and policy makers need to work together to address the organisational, community and policy barriers to healthy eating.

## Figures and Tables

**Table 1:** Comparison of sociodemographic characteristics, medical history and dietary habits for those reported meeting and not meeting My Healthy Plate recommendation (excluding those on special diet).

Characteristic ^#^	Did not Meet the Recommendation (*n* = 175)	Met the Recommendation (*n* = 248)	*p* Value ^+^
**Sociodemographic characteristics**			
Sex			
Female	82 (46.9)	120 (48.4)	0.76
Male	93 (53.1)	128 (51.6)	
Ethnicity			
Chinese	136 (77.7)	205 (82.7)	0.51
Malay	25 (14.3)	24 (9.7)	
Indian	12 (6.9)	16 (6.4)	
Others	2 (1.1)	3 (1.2)	
Marital status			
Single	34 (19.4)	26 (10.5)	0.009
Married	141 (80.6)	222 (89.5)	
Highest education level			
No formal education	7 (4.0)	10 (4.1)	1.00
Primary	47 (26.9)	67 (27.0)	
Secondary	73 (41.7)	102 (41.1)	
Post-secondary	48 (27.4)	69 (27.8)	
Housing type *			
1–3 room public housing	30 (17.2)	49 (19.8)	0.80
4–5 room public housing	107 (61.5)	147 (59.5)	
Executive flat/private property	37 (21.3)	51 (20.7)	
Current work status			
Currently working	108 (61.7)	131 (52.8)	0.07
Not working	67 (38.3)	117 (47.2)	
Age in years, mean (SD)	60.4 (9.3)	63.0 (7.9)	0.003
**Medical history**			
Type of prediabetes			
Impaired fasting glycaemia	90 (51.4)	135 (54.4)	0.54
Impaired glucose tolerance	85 (48.6)	113 (45.6)	
Years with prediabetes, mean (SD)	2.0 (2.0)	2.1 (2.3)	0.54
**Dietary habits**			
Frequency of breakfast			
Almost everyday	153 (87.4)	226 (91.1)	0.47
Sometimes	14 (8.0)	14 (5.7)	
Rarely or never	8 (4.6)	8 (3.2)	
Frequency of main meals daily, median (IQR) *	3.0 (2.0–4.0)	3.0 (2.0–3.0)	0.63
Frequency of snacks daily, median (IQR)	1.0 (0–3.0)	1.0 (0–3.0)	0.55
Frequency of eating out weekly, mean (SD)	5.9 (5.7)	4.2 (3.7)	<0.001
Frequency of deep-fried food consumption weekly, mean (SD)	2.1 (1.7)	1.4 (1.4)	<0.001
Frequency of sweet desserts weekly, mean (SD)	1.3 (1.7)	1.1 (1.4)	0.16

All figures in the table referred to frequency (column percentage) unless otherwise indicated; * Contained missing numbers (housing type, 2; and frequency of main meals daily, 4); ^+^ The p-values were computed using χ2 test or Fisher Exact test (whichever appropriate) for categorical variables, ordinal variables with the Mann–Whitney U test, and two-sample t-test for continuous variables; ^#^ Out of 433 participants, 10 were on special diet and excluded from the statistical analysis. IQR, interquartile range referred to the range of values of a frequency distribution between the 25th and 75th percentiles. SD referred to standard deviation.

**Table 2:** Crude and adjusted prevalence ratio (PR) of sociodemographic characteristics, medical history and dietary habits associated with meeting My Healthy Plate recommendation (excluding those on special diet).

Characteristic ^#^	Crude PR (95% CI)	*p* Value	Adjusted PR ^+^ (95% CI)	*p* Value
**Sociodemographic characteristics**				
Sex				
Female	Referent	0.78	Referent	0.23
Male	0.97 (0.82–1.16)		1.11 (0.93–1.32)	
Ethnicity				
Chinese	Referent	<0.001	Referent	0.03
Malay	0.81 (0.74–0.89)		0.81 (0.70–0.95) ^#^	
Indian	0.95 (0.56–1.61)		0.89 (0.51–1.54)	
Others	1.00 (0.51–1.95)		0.98 (0.41–2.30)	
Marital status				
Single	Referent	<0.001	Referent	0.001
Married	1.41 (1.18–1.69)		1.38 (1.13–1.68) ^#^	
Highest education level				
No formal education	Referent	0.99	Referent	0.17
Primary	1.00 (0.61–1.63)		1.14 (0.70–1.87)	
Secondary	0.99 (0.69–1.43)		1.22 (0.81–1.85)	
Post-secondary	1.00 (0.71–1.42)		1.24 (0.84–1.81)	
Housing type *				
1–3 room public housing	Referent	0.09	Referent	0.06
4–5 room public housing	0.93 (0.85–1.02)		0.90 (0.78–1.03)	
Executive flat/private property	0.93 (0.76–1.15)		0.88 (0.75–1.04)	
Current work status				
Currently working	Referent	0.11	Referent	0.97
Not working	1.16 (0.97–1.39)		1.00 (0.83–1.19)	
Age in years	1.01 (1.01–1.02)	0.001	1.01 (1.00–1.01)	0.16
**Medical history**				
Type of prediabetes				
Impaired fasting glycaemia	Referent	0.53	Referent	0.44
Impaired glucose tolerance	0.95 (0.81–1.11)		0.94 (0.81–1.10)	
Years with prediabetes	1.01 (0.99–1.03)	0.34	1.00 (0.98–1.03)	0.83
**Dietary habits**				
Frequency of breakfast				
Almost everyday	Referent	0.28	Referent	0.42
Sometimes	0.84 (0.64–1.09)		0.86 (0.68–1.09)	
Rarely or never	0.84 (0.45–1.55)		0.93 (0.51–1.69)	
Frequency of main meals daily *	0.99 (0.80–1.22)	0.94	0.96 (0.78–1.17)	0.66
Frequency of snacks daily	0.97 (0.87–1.08)	0.59	0.99 (0.89–1.09)	0.82
Frequency of eating out weekly	0.96 (0.95–0.98)	<0.001	0.97 (0.96–0.98) ^#^	<0.001
Frequency of deep-fried food consumption weekly	0.88 (0.81–0.95)	0.001	0.90 (0.82–0.98) ^#^	0.02
Frequency of sweet desserts weekly	0.96 (0.91–1.02)	0.18	1.00 (0.97–1.04)	0.95

* Contained missing numbers (housing type, 2; and frequency of main meals daily, 4). ^+^ The adjusted PR (aPR) of the variables that were not significant at the 5% level was obtained by incorporating that particular variable in the final multivariable model. ^#^ These variables were significant at the 5% level and were included in the final multivariable model using the backward stepwise approach. ^#^ Out of 433 participants, 10 were on special diet and excluded from the statistical analysis.

**Table 3:** Participant characteristics for the in-depth interviews

Characteristic	*n* = 48
**Sociodemographic characteristics**	
Sex	
Female	24 (50.0)
Male	24 (50.0)
Ethnicity	
Chinese	37 (77.1)
Malay	6 (12.5)
Indian	5 (10.4)
Age in years, mean (SD)	59.8 (9.1)
**Behaviour**	
Healthy plate recommendation	
Meeting	24 (50.0)
Not meeting	24 (50.0)

All figures in the table referred to frequency (column percentage) unless otherwise indicated

## Supplementary Materials

The following are available online at https://www.mdpi.com/2072-6643/11/5/1014/s1, File S1: A survey on pre-diabetes seeking care at the polyclinic.
