# Supplementary material for: Correlates, Facilitators and Barriers of Healthy Eating Among Primary Care Patients with Prediabetes in Singapore—A Mixed Methods Approach
Source: Nutrients. 2019 May 6;11(5):1014. doi: 10.3390/nu11051014 (PMC6566398; doi:10.3390/nu11051014)
Supplement: Supplementary file 1 [file nutrients-11-01014-s001.pdf]

Date: ________________

Time: ________________

Survey ID: _____________

NUS National University of Singapore | Saw Swee Hock School of Public Health

National Healthcare Group POLYCLINICS Advancing Family Medicine, Transforming Primary Healthcare

A survey on pre-diabetes seeking care at the polyclinic

The information gathered for this survey will be used to develop a health promotion programme for patients like you. There is no correct or wrong answer. Please tick (√) the box that is the most appropriate for you.

Section 1: Sociodemographic Data

1	**Citizenship Status**	☐ Singaporean ☐ Permanent Resident
2	**Year of Birth**	________________
3	**Gender**	☐ Male ☐ Female
4	**Ethnicity**	☐ Chinese ☐ Malay ☐ Indian<br>☐ Others (please specify): ______________________________
5	**Current Marital Status**	☐ Never married ☐ Currently married<br>☐ Separated ☐ Divorced<br>☐ Widowed
6	**Highest Education Level**	☐ No formal education ☐ Primary<br>☐ Secondary ☐ Polytechnic/ITE<br>☐ Junior College ☐ University/Postgraduate<br>☐ Others: ____________________________________
7	**Current work status**	☐ Full-time work, specify occupation: ________________________<br>☐ Part-time work, specify occupation: ________________________<br>☐ Homemaker/Housewife<br>☐ Retired, specify previous occupation: ______________________<br>☐ Unemployed (unable to work because of disability or other medical conditions)<br>☐ Unemployed (able to work), specify previous occupation:<br>____
8	**Monthly Household Income Over the Last Year**	☐ Below $2,000 ☐ $2,000 - $3,999 ☐ $4,000 - $5,999<br>☐ $6,000 - $9,999 ☐ $10,000 and above<br>☐ Do not know

9a	**Housing Type**	☐ HDB 1-room ☐ HDB 2-room ☐ HDB 3-room<br>☐ HDB 4-room ☐ HDB 5-room ☐ HUDC Flat<br>☐ HDB/Government Executive Flat<br>☐ Private Apartment/Condominium<br>☐ Terrace/Semi-detached House/Bungalow<br>☐ Others (please specify): ________________
9b	**Housing Ownership**	☐ Owned ☐ Rental

Section 2: Medical and Family History

	(i) Have you ever been told by a doctor (Western trained) that you have the following conditions?	(ii) If you answered 'Yes' to (i), for how many years have you had the condition?	(iii) Are you currently on follow-up with a doctor (Western trained) for the condition?
10a. High blood pressure	☐ Yes ☐ No	____________ years	☐ Yes ☐ No
10b. High blood cholesterol or lipids	☐ Yes ☐ No	____________ years	☐ Yes ☐ No
10c. Stroke	☐ Yes ☐ No	____________ years	☐ Yes ☐ No
10d. Heart disease	☐ Yes ☐ No	____________ years	☐ Yes ☐ No
10e. Kidney disease	☐ Yes ☐ No	____________ years	☐ Yes ☐ No
10f. Gout	☐ Yes ☐ No	____________ years	☐ Yes ☐ No
10g. Asthma	☐ Yes ☐ No	____________ years	☐ Yes ☐ No
10h. Cancer, specify site____________	☐ Yes ☐ No	____________ years	☐ Yes ☐ No
10i. Others not listed above, please specify ____________	☐ Yes ☐ No	____________ years	☐ Yes ☐ No
**If you are a female, please answer whether you have the following conditions.**			
10j. Gestational diabetes (high blood sugar during pregnancy)	☐ Yes ☐ No	____________ years	☐ Yes ☐ No
10k. Polycystic ovary syndrome (hormonal imbalance leading to several cysts in ovaries)	☐ Yes ☐ No	____________ years	☐ Yes ☐ No

Now I would like to ask questions on your pre-diabetes (higher than normal blood sugar but not high enough to be diabetes)

Oral glucose tolerance test (OGTT) involves 2 blood sugar tests, one time after 8 hours of fasting in the morning and then another time at 2 hours later after you consume a sugary drink.

11a. What type of pre-diabetes do you have?

☐ Impaired fasting glycaemia (<7.8 mmol/L on 2-hour post OGTT)
☐ Impaired glucose tolerance (7.8 – 11.0 mmol/L on 2-hour post OGTT)
☐ Not sure (Go to the next page Q12a)

11b. For how long have you had pre-diabetes?

___________ year(s) ___________ month(s)

11c. Other than seeing the doctor here, do you seek consultation elsewhere for your pre-diabetes?

☐ No
☐ Yes, other polyclinic or GP clinic
☐ Yes, specialist clinic in public or private hospital
☐ Yes, Traditional Chinese Medicine (TCM) clinic
☐ Yes, other not listed above, please specify, ________________________________

12a. Has anyone else in your family/friends ever had pre-diabetes?

☐ Yes (go to Q12b)
☐ No (go to the next page Q13a)
☐ Do not know (go to the next page Q13a)

[If "Yes" to Q12a]

Q12b	Can you tell me who in your family/circle of friends has had pre-diabetes?	Is this person currently staying with you in the same house?
i. Father	☐ Yes ☐ No ☐ Do not know	☐ Yes ☐ No
ii. Mother	☐ Yes ☐ No ☐ Do not know	☐ Yes ☐ No
iii. Brother	☐ Yes ☐ No ☐ Do not know ☐ Not applicable	☐ Yes ☐ No
iv. Sister	☐ Yes ☐ No ☐ Do not know ☐ Not applicable	☐ Yes ☐ No
v. Son	☐ Yes ☐ No ☐ Do not know ☐ Not applicable	☐ Yes ☐ No
vi. Daughter	☐ Yes ☐ No ☐ Do not know ☐ Not applicable	☐ Yes ☐ No
vii. Friend(s)	☐ Yes ☐ No ☐ Do not know ☐ Not applicable	☐ Yes ☐ No
viii. Others, specify________	☐ Yes ☐ No ☐ Do not know ☐ Not applicable	☐ Yes ☐ No

12c. To what extent have your following behaviours changed because your family member/friend(s) also had pre-diabetes?

Please tick (√) **ONLY ONE** option for **EACH ROW**.

**Behaviour**	**1**<br>**No changes at all**	**2**<br>**To a small extent**	**3**<br>**To a moderate extent**	**4**<br>**To a large extent**	**5**<br>**To a very large extent**
Eating more healthily					

**Behaviour**	**1**<br>**No changes at all**	**2**<br>**To a small extent**	**3**<br>**To a moderate extent**	**4**<br>**To a large extent**	**5**<br>**To a very large extent**
Being more physically active					
Going for screening regularly					
Others, specify________________					

13a. Has anyone in your family/circle of friends ever had diabetes?

☐ Yes (go to Q13b)
☐ No (go to Section 3)
☐ Do not know (go to Section 3)

Q13b	Can you tell me who has had diabetes?	Is this person currently staying with you in the same house?
i. Father	☐ Yes ☐ No ☐ Do not know	☐ Yes ☐ No
ii. Mother	☐ Yes ☐ No ☐ Do not know	☐ Yes ☐ No
iii. Brother	☐ Yes ☐ No ☐ Do not know ☐ Not applicable	☐ Yes ☐ No
iv. Sister	☐ Yes ☐ No ☐ Do not know ☐ Not applicable	☐ Yes ☐ No
v. Son	☐ Yes ☐ No ☐ Do not know ☐ Not applicable	☐ Yes ☐ No
vi. Daughter	☐ Yes ☐ No ☐ Do not know ☐ Not applicable	☐ Yes ☐ No
vii. Friend(s)	☐ Yes ☐ No ☐ Do not know ☐ Not applicable	☐ Yes ☐ No
vii. Others, specify__________	☐ Yes ☐ No ☐ Do not know ☐ Not applicable	☐ Yes ☐ No

13c. To what extent have your following behaviours changed because your family member/friend(s) had diabetes? Please tick (√) **ONLY ONE** option for **EACH ROW**.

**Behaviour**	**1**<br>**No changes at all**	**2**<br>**To a small extent**	**3**<br>**To a moderate extent**	**4**<br>**To a large extent**	**5**<br>**To a very large extent**
Eating more healthily					
Being more physically active					
Going for screening regularly					
Others, specify________________					

Section 3: Dietary habits

14. How often do you eat breakfast?	15. How many main meals do you eat each day?	16. How often do you snack each day in between your main meals?
☐ Almost every day (at least 5 days a week)<br>☐ Sometimes (2 to 4 days a week)<br>☐ Rarely or never (one or less days a week)	_________ each day	_________ each day

17. How often do you eat at hawker centres, food courts or coffee shops in a typical week?	18. How often do you eat at fast food restaurants (e.g. KFC, McDonald's etc.) in a typical week?
_________times per week	_________ times per week

19. Where do you **USUALLY** eat the following meals?

Please tick (√) **ONLY ONE** option for **EACH COLUMN**.

a. Breakfast	b. Lunch	c. Dinner
☐ Home	☐ Home	☐ Home
☐ Packed from home	☐ Packed from home	☐ Packed from home
☐ Workplace canteen	☐ Workplace canteen	☐ Workplace canteen
☐ Hawker centre/Coffee shop/Food court	☐ Hawker centre/Coffee shop/Food court	☐ Hawker centre/Coffee shop/Food court
☐ Fast food restaurant	☐ Fast food restaurant	☐ Fast food restaurant
☐ Others, specify ______________________	☐ Others, specify ______________________	☐ Others, specify ______________________
☐ Do not eat breakfast at all	☐ Do not eat lunch at all	☐ Do not eat dinner at all

20a. What type of rice do you **USUALLY** eat for your main meal?	20b. How many bowls of rice do you **USUALLY** eat for your main meal?
☐ White rice<br>☐ Basmati rice<br>☐ Brown or red rice<br>☐ Mixture of white and brown or red rice<br>☐ Others, specify ____________________<br>☐ Do not eat rice at all	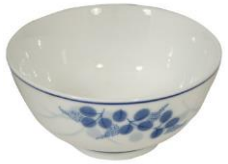<br>_______ bowls per main meal

20c. On occasions that you do not eat rice, what do you **USUALLY** consume as your alternative staple? You may select more than 1 option.

☐Breads (Wholemeal/Wholegrain/High fibre bread)
☐Breads (Non-Wholemeal/Wholegrain/High fibre bread)
☐Oats/Muesli
☐Starchy vegetables (e.g. Potatoes, Yam)
☐Whole grain noodles (e.g. Brown rice vermicelli, Buckwheat noodles)
☐Non-whole grain noodles (e.g. Yellow noodles, Rice Bee Hoon, Kway Teow)
☐Pasta (Wholemeal/Wholegrain)
☐Pasta (Non-Wholemeal/Non-Wholegrain)
☐Others, specify:______________________
☐Do not eat rice alternatives at all

21. How many days a week do you eat legumes (e.g. Lentils, Chickpeas, Red/Green/Soya beans)?

☐ Almost every day (at least 5 days a week)
☐ Sometimes (two to four days a week)
☐ Rarely or never (one or less days a week)

22. How often do you consume the following in a typical week?

a. **Deep fried foods** (E.g. fried chicken, fried fish, French fries etc.)	b. **Sweet desserts and snacks** (E.g. cakes, kuehs, jellies, candies, chocolates, cookies and ice-cream etc.)
_________times per week	_________times per week

23. How many servings of vegetables do you eat on a typical day?
(Please refer to the chart below on the references for servings of vegetables. You may ask for a bigger picture from our research assistant.)

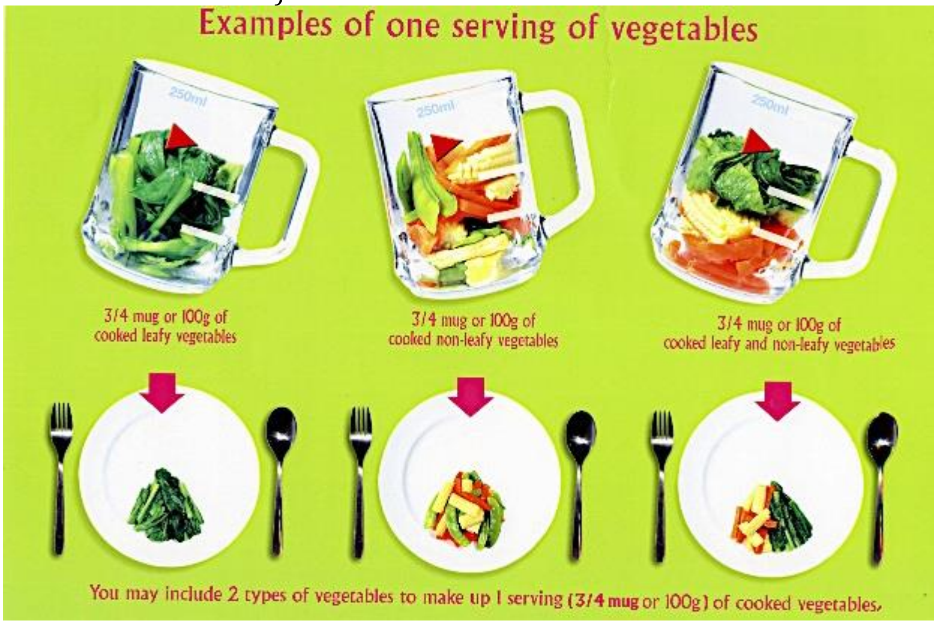

If you don't usually eat vegetables on a typical day, please indicate "0".

__________ servings on a typical day

24. Excluding fresh juices, how many servings of fresh fruit do you eat on a typical day?
(Please refer to the chart below on the references for servings of fruits)

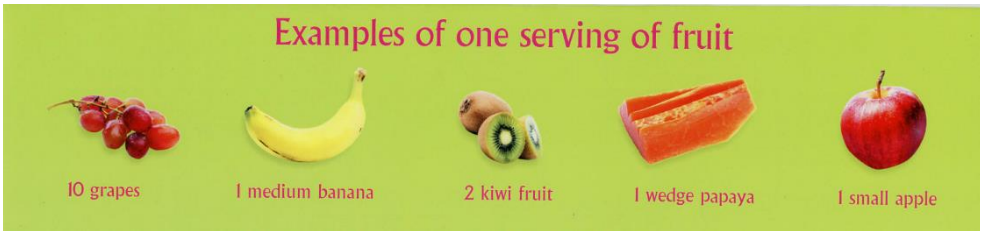

If you don't usually eat fresh fruits on a typical day, please indicate "0".

__________ servings on a typical day

25. How often do you consume the following beverages on a typical day?

If you don't drink this beverage at all in a day, please indicate "0"

a. Coffee (hot or cold)	b. Tea (hot or cold)	c. Sweetened drinks (E.g. soft drinks, packet drinks, packet fruit drinks, yoghurt-based drinks and cultured milk drinks etc.)
__________times a day	__________times a day	__________times a day

26. How often do you ask for "less sugar"/"no sugar (kosong)" when ordering the following beverages?	
a. Coffee (hot or cold)	b. Tea (hot or cold)
☐ Often or always<br>☐ Sometimes<br>☐ Rarely or never	☐ Often or always<br>☐ Sometimes<br>☐ Rarely or never

27. How often do you choose "reduced sugars"/"No added sugar"/"sugar–free" when buying the SWEETENED beverages (e.g. soft drinks, packet drinks, packet fruit drinks, yoghurt-based drinks and cultured milk drinks etc.?

☐ Often or always
☐ Sometimes
☐ Rarely or never

28. When you eat meat with visible fat, how much visible fat will you trim off?	29. When you eat poultry (e.g. chicken, duck, turkey etc.), how much skin do you remove?
☐ All the fat<br>☐ Some of the fat<br>☐ None of the fat<br>☐ Do not eat meat at all	☐ All the skin<br>☐ Some of the skin<br>☐ None of the skin<br>☐ Do not eat poultry at all

30. At the table, do you add salt or sauces to your food?

☐ Usually
☐ Sometimes
☐ Very occasionally
☐ Never

31. Which of the following **BEST** describes your **CURRENT** diet?
Please tick (√) **ONLY ONE** answer.

☐ I am on a vegetarian/vegan diet
☐ I am on a weight loss diet, specify diet: ____________________
☐ I am on a fat modified diet, specify diet: ____________________
☐ Other not listed above, specify diet: ____________________
☐ I am NOT on any special diet

32. Based on your **CURRENT** diet on a **TYPICAL** day, are you able to fulfil the following requirements of 'My Healthy Plate' recommended by the Health Promotion Board (HPB)?

This means in my daily diet

- About **half** consists of fruits and vegetables
- About **a quarter** consists of whole grains such as brown rice, red rice, wholemeal bread or rolled oats
- About **a quarter** consists of meat and others (seafood, eggs, nuts, bean products and dairy products)

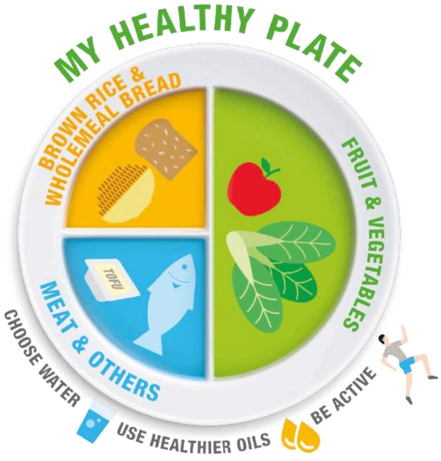

☐ Yes (go to Section 4)
☐ No (go to Q33)
☐ Not sure (go to Q33)
☐ Not applicable, as I am on special diet (e.g. vegetarian/vegan diet, weight loss diet or fat-modified diet etc.) (go to Section 4)

33. To what extent do the following reasons prevent you from fulfilling the requirements of 'My Healthy Plate' (i.e. healthy and balanced diet) recommended by the HPB?

Please tick (√) **ONLY ONE** option for **EACH ROW**.

**I cannot fulfil the requirements of 'My Healthy Plate' because of the following reasons.**	**1 Strongly disagree**	**2 Somewhat disagree**	**3 Neutral**	**4 Somewhat agree**	**5 Strongly agree**
Lack of interest in having a healthy and balanced diet					
Lack of self-motivation to have a healthy and balanced diet					
Lack of time					
Lack of energy (Too tired)					
Lack of knowledge on how to have a healthy and balanced diet					
Lack of skills on how to choose healthier food options					
Lack of skills on how to prepare healthier home-cooked food					
Healthier food is not tasty to me					
I cannot resist the temptation of unhealthy food such as fast food etc.					
I have no control over home-cooked food and I eat whatever is being cooked					
My family likes to eat unhealthy food and I have to eat with them					
My friends like to eat unhealthy food and I eat with them to socialise					
My work does not allow me to have enough time or opportunities for healthier meals					
Healthier food is more expensive in the workplace canteen					
My workplace canteen does not offer healthier food options					
Healthier food is more expensive in the hawker centre/coffee shop/ food court that I go to					
The hawker centre/coffee shop/ food court that I go to does not offer healthier food options					

Section 4: Contact details

We would be holding one-to-one interviews to help us better understand the motivations and self-care skills required for pre-diabetics like you to adopt a healthy lifestyle. This would take place for an hour at National University of Singapore. Participants will be reimbursed $20 for their time. Please kindly leave your contact details if you wish to be invited for the interview. All information will be kept strictly confidential.

Preferred name: ____________________________ Contact number: ____________________________

Email: _____________________________ Spoken languages: English/Chinese/Malay

Preferred day and time:

Weekdays	Weekends
☐ Morning<br>☐ Early afternoon<br>☐ Late afternoon	☐ Morning<br>☐ Early afternoon<br>☐ Late afternoon

THANK YOU
